# Hemolysis, Elevated Liver Enzymes and Low Platelets (HELLP) Syndrome and Metastatic Breast Cancer: A Rare Overlap in a Postpartum Patient With Disseminated Intravascular Coagulation and Refractory Thrombocytopenia

**DOI:** 10.7759/cureus.42225

**Published:** 2023-07-20

**Authors:** Elvis Mesa, Dylan Miles, Nayle Araguez-Ancares, Damian Casadesus

**Affiliations:** 1 Internal Medicine, St. George's University School of Medicine, St. George’s, USA; 2 Internal Medicine, Jackson Memorial Hospital, Miami, USA; 3 Internal Medicine, St. George's University School of Medicine, St. George's, GRD

**Keywords:** deep vein thrombosis (dvt), acute subdural hematoma, disseminated intravascular coagulation, hellp syndrome, metastatic breast cancer, refractory thrombocytopenia

## Abstract

A middle-aged female arrived at a tertiary care hospital after a referral from her primary care physician to evaluate a left breast mass found on ultrasound concerning malignancy. The patient was also 27 weeks gestational with monochorionic diamniotic twins. During triage, she was found to have severe hypertension and lab abnormalities concerning HELLP (hemolysis, elevated liver enzymes, low platelets) syndrome and underwent cesarean delivery of the infants. She had a biopsy of the left breast mass post-delivery, later diagnosed as invasive ductal cell carcinoma with spinal metastasis and numerous metastatic pulmonary nodules. Her hospital stay was complicated by a right lower extremity deep vein thrombosis, acute subdural hematoma, and disseminated intravascular coagulation with refractory thrombocytopenia resulting in her death.

## Introduction

Disseminated Intravascular Coagulation (DIC) is a condition marked by systemic activation of coagulation and consumption of clotting factors, leading to thrombosis and or bleeding. It may result in a complication of infection, trauma, aneurysms, liver diseases, obstetric disorders, and malignancy [1]. In patients with cancer, the pathogenic mechanism of DIC is not clearly understood. A possible mechanism is the expression of tumor factors which can activate coagulation factors or by expression of fibrinolytic proteins [2]. We present a case of a pregnant patient who was admitted for HELLP syndrome and developed postpartum DIC, complicated by refractory thrombocytopenia, with her workup revealing an underlying malignancy.

## Case presentation

A 42-year-old woman with monochorionic diamniotic twins at twenty-seven weeks gestational age was emergently admitted because of hypertension (178/110 mmHg), anemia, and transaminitis secondary to HELLP syndrome. The patient's laboratory reports were significant for a hemoglobin of 9.1 g/dl, an alanine aminotransferase of 68 U/L, an aspartate aminotransferase of 137 U/L, and a platelet count of 41 x 10^3^ mcL. She complained of progressively worsening, constant lower back and bilateral thigh pain. Upon triage, it was noted that she had asymmetrical breast tissue with a predominant solid and irregular left breast mass of approximately 15 x 10 cm and marked left axillary lymphadenopathy. She also had hardened areolar tissue and noticeable nipple retraction. Because her condition suggested the presence of hematologic disease associated with pregnancy, the patient was recommended to undergo an urgent cesarean delivery of the twins as this is the only effective treatment for HELLP syndrome. Cesarean delivery was performed under general endotracheal anesthesia to mitigate any possible spinal or epidural hematoma risks associated with neuraxial anesthesia. The patient was scheduled to receive a platelet transfusion during the delivery with possible packed red blood cells and fresh frozen plasma. Despite receiving one unit of thawed apheresis platelets during the cesarean, the patient's platelet count minimally improved to 50 x 10^3^ mcL.

**Figure 1:**
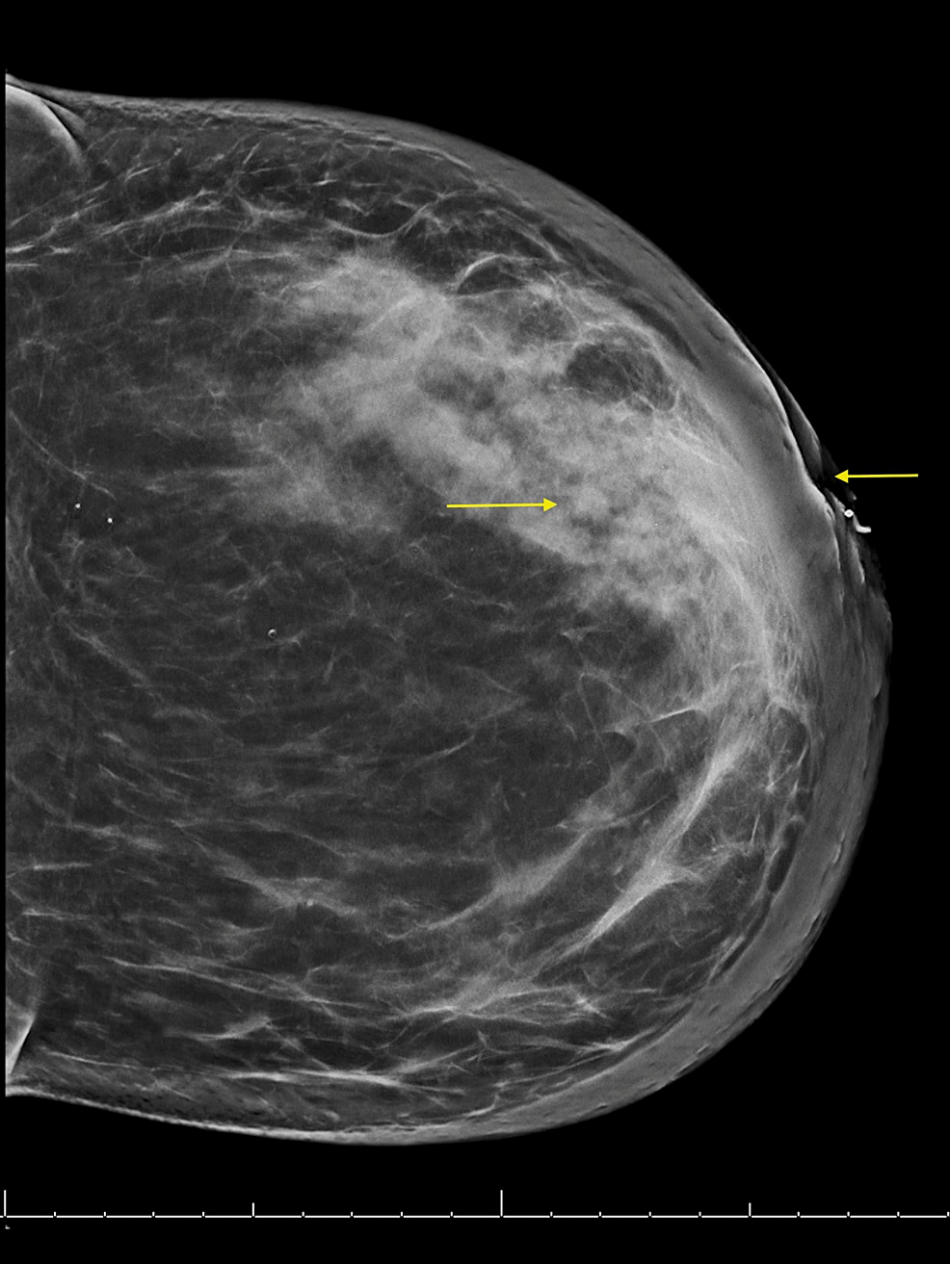
Diagnostic mammogram The diagnostic mammogram revealed diffuse skin and trabecular thickening of the left breast with associated left nipple retraction (yellow arrows).

The patient underwent a diagnostic mammogram of the left breast mass, which revealed findings consistent with diffuse thickening of the left breast with associated left nipple retraction (Figure 1). These findings highly suggested malignancy, warranting a left breast and axillary lymph node biopsy. Findings of these biopsies were consistent with poorly differentiated, estrogen receptor (ER)/progesterone receptor (PR) negative, HER2/neu positive, Ki-67 expressing invasive ductal cell carcinoma.

**Figure 2:**
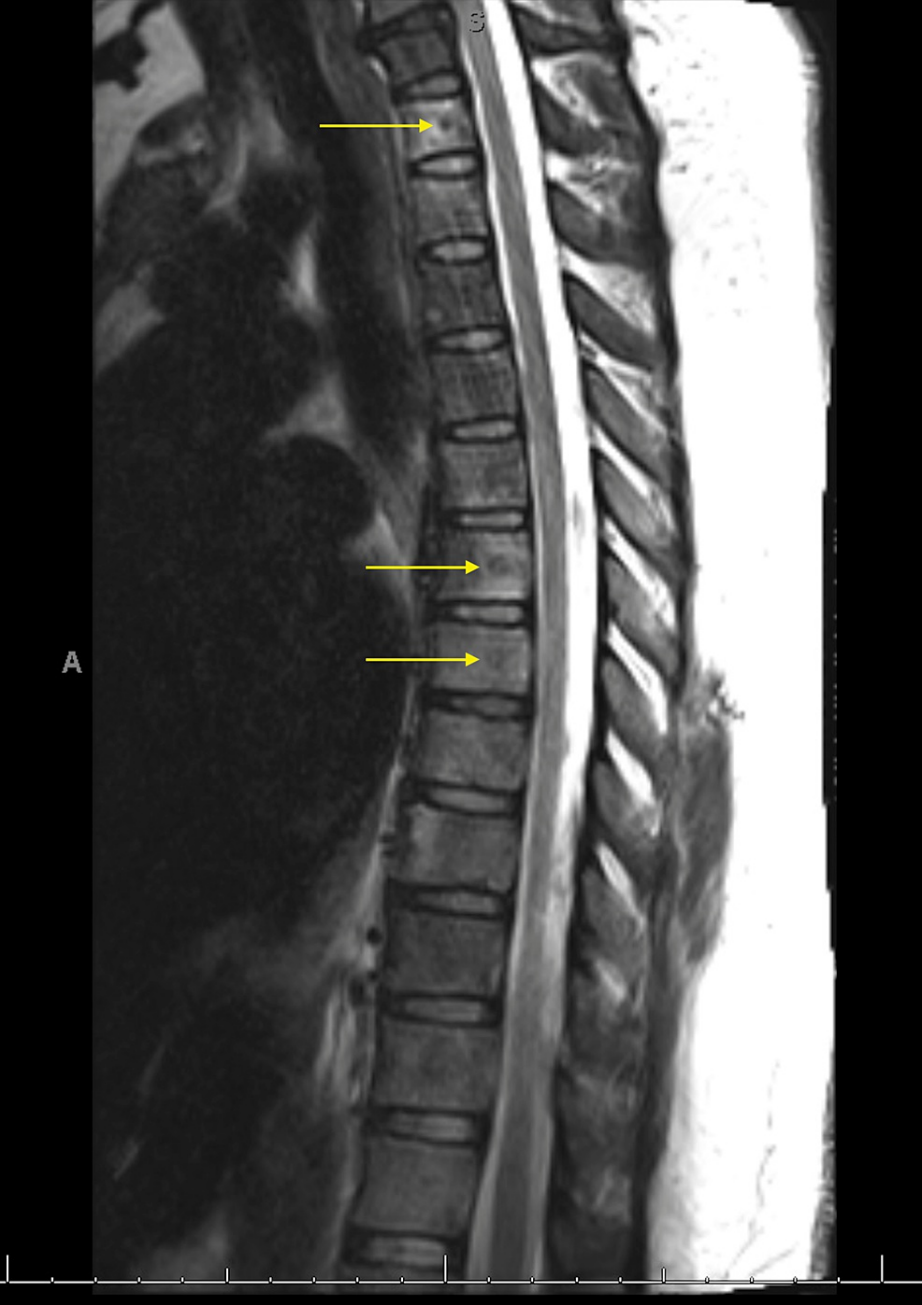
MRI of the spine MRI of the spine revealed multilevel metastatic disease in the thoracic spine.

Given the diagnosis of invasive ductal cell carcinoma, the patient underwent a chest CT and an MRI of the cervical and thoracic spine. Results of the CT of the chest revealed numerous pulmonary nodules in a random distribution, likely indicative of metastasis. Results of the spinal MRI revealed multilevel metastatic disease in the thoracic spine with no associated compression fractures, canal, or foramina involvement (Figure 2).

**Figure 3:**
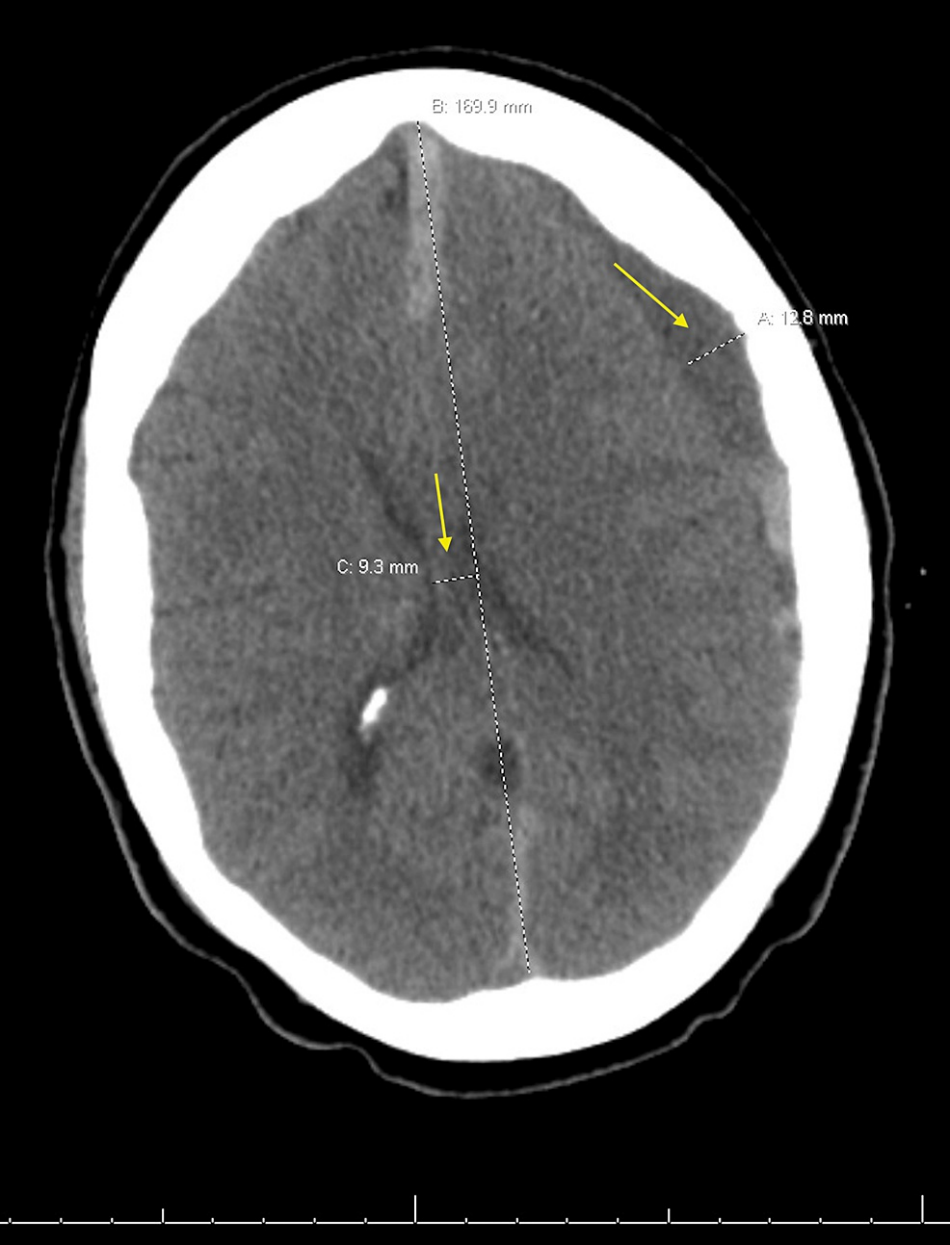
CT scan image of the head CT of the head revealed a 12.8 mm left subdural hematoma with a 9.3 mm midline shift to the right (yellow arrows).

On the tenth day of admission, the patient developed a right lower extremity deep vein thrombosis (DVT), requiring anticoagulation with heparin. Two days later, she experienced left-sided numbness, left gaze palsy, and severe headache. She was diagnosed with a left subdural hematoma with a midline shift to the right (Figure 3). Neurosurgery recommended stopping anticoagulation and undergoing left bifrontal burr holes for subdural hematoma evacuation. Venous thromboembolism prophylaxis was achieved by placing an inferior vena cava (IVC) filter.

Hematology and oncology specialties were consulted due to complicated disseminated intravascular coagulation (DIC) secondary to metastatic breast cancer. Although urgent chemotherapy was needed to treat the underlying cause of DIC, the patient was no longer a candidate based on her critical hematological condition. Peripheral blood smear showed findings consistent with normocytic, normochromic red blood cells and schistocytes. At the time of the hematology consult, her laboratory results were significant for anemia, severe thrombocytopenia, abnormal coagulation panel, and elevated D-dimers >20 mcg/mL (Table 1). 

**Table 1:** Relevant laboratory results upon investigation WBC- white blood cell, RBC- red blood cell, aPTT- activated partial thromboplastin time, AST- aspartate aminotransferase, ALT- alanine aminotransferase

Test	Patient’s value	Normal reference
WBC	13.4 x 10^3^/uL	3.8-10.8
Hemoglobin	6.3 g/dL	13.2-17.1
RBC	1.98 x 10^6^/uL	4.20-5.80
Hematocrit	18.6 %	38.5-50.0
Platelet count	7 x 10^3^/uL	140-400
D-dimers	>20 µg/mL	<0.50
Fibrinogen	62 mg/dL	175-425
Prothrombin Time	18 sec	10-13
aPTT	55 sec	30-40
AST	120 U/L	10-40
ALT	147 U/L	9-46

During her extended stay in the hospital, the patient required supportive therapy with multiple blood products, mostly platelet transfusions (total of 55), which proved unsuccessful in raising the platelet count. Based on refractory thrombocytopenia and past medical history of low platelets, immune thrombocytopenia (ITP) was considered after ruling out potential causes such as HIV and hepatitis C. It was recommended to start 40 mg intravenous dexamethasone for four days, intravenous immune globulins daily for two days, and weekly romiplostim starting at 2 mcg/kg and titrated to 3 mcg/kg. Nonetheless, the platelet count failed to respond, and romiplostim was discontinued after four weeks.

On day 45 of admission, our patient developed encephalopathy due to intracerebral hemorrhage and respiratory distress secondary to pulmonary edema in the setting of multiple transfusions. She was intubated and placed on mechanical ventilation, which resulted in pulseless electrical activity and cardiac arrest. Fortunately, spontaneous circulation was returned after four minutes of advanced cardiovascular life-sustaining measures. The following day, the patient became severely hypotensive, likely secondary to hemorrhagic shock, requiring norepinephrine. It was decided to administer epsilon-aminocaproic acid (EACA) 5 grams over 60 minutes due to persistent bleeding from the burr hole incision site, epistasis, and new onset melena. Nonetheless, despite aggressive fluid resuscitation, vasoactive drips, and several units of blood products, the patient succumbed to the illness and was pronounced deceased.

## Discussion

This case report illustrates a rare overlapping of two potential triggers for DIC: HELLP syndrome and malignancy. Nonetheless, based on the insidious stage 4 (metastatic) breast cancer and past medical history of thrombocytopenia (before pregnancy), we considered cancer-related DIC the primary point for this discussion. 

When dealing with patients with cancer-related DIC, it is helpful to consider the pathogenetic mechanisms that can lead to thrombosis and bleeding. The procoagulant and hyperfibrinolytic states in DIC can manifest simultaneously, which can be challenging for medical management [3]. For instance, these two states were evidenced in our patient when she had a right lower extremity deep vein thrombosis requiring anticoagulation. Furthermore, she developed a subdural hematoma, intracranial bleeding, gastrointestinal bleeding, and hemorrhagic shock, requiring placement of an inferior vena cava filter and holding heparin. A negative serotonin release assay ruled out heparin-induced thrombocytopenia. Although our patient's initial routine coagulation studies (prothrombin time {PT}/international normalized ratio {INR}, partial thromboplastin time {PTT}, fibrinogen, and D-Dimers) were consistent with DIC, it is crucial to recognize that these tests are not specific and have poor sensitivity. In the setting of DIC, elevated D-dimer can be more sensitive to monitor disease progression [4]. Elevated serum D-dimers are a direct consequence of fibrinolysis and, thus, direct indicators of increased thrombotic activity such as DIC and venous thromboembolism [5]. Our patient D-dimers remained consistently elevated during admission (>20.00) without returning to normal values.

The medical management of DIC is very complex, aiming to target the underlying disease processes. In this case, the patient presented with overlaying triggers: HELLP syndrome and stage 4 breast cancer. Urgent delivery was performed as this is the only effective treatment for HELLP syndrome. Although urgent chemotherapy was needed to treat the metastatic breast cancer, she was not a good candidate. The initiation of chemotherapy would likely have worsened the thrombocytopenia resulting in catastrophic hemorrhage. 

It is worth noting our patient's metastatic breast cancer, which exacerbated her hematological state by bone marrow infiltration, disrupting normal hematopoiesis. Her biopsies were consistent with poorly differentiated, ER/PR negative, HER2/neu positive, Ki-67 expressing invasive ductal cell carcinoma. According to a meta-analysis and systematic review, breast cancer in pregnancy is marked by a low hormone receptor-positive, a higher Ki-67 nuclear antigen index, and overexpression of HER2 [6]. This meta-analysis showed that 73.7% of patients were hormone receptor-negative and presented with a more invasive stage than non-pregnant patients.

Since early admission, our patient presented with refractory thrombocytopenia (ranging from 7 to 45 thousand/uL), requiring a total of 55 platelet transfusions during hospitalization. From the clinical hematological viewpoint, we decided that supportive care with packed red blood cells, cryoprecipitate, fresh frozen plasma (FFP), and platelet transfusions was the most appropriate medical management. Based on her consistently low fibrinogen (ranging between 62 mg/dL and 306 mg/dL), cryoprecipitate provided a good source of fibrinogen with significantly less volume load than FFP. Nevertheless, she developed pulmonary edema secondary to fluid overload in the setting of blood product transfusions and aggressive fluid resuscitation.

The presumptive diagnosis of primary ITP was considered after ruling out potential etiologies for thrombocytopenia, such as HIV and hepatitis C. On review of laboratory results (before pregnancy), she presented with low platelets (57 to 73 x 10^3 ^mcL). She denied bleeding or bruising at that time. Since thrombocytopenia may be induced by cancer-related DIC or ITP, selecting a treatment for such a patient could be difficult. We chose to use intravenous immunoglobulin (IVIG) and glucocorticoids together. According to the medical literature, IVIG can raise the platelet count within 1 to 3 days and glucocorticoids within 2 to 14 days. Regarding second-line therapy, romiplostim is a thrombopoietin receptor agonist that stimulates the production of megakaryocytes and, ultimately, platelets in the bone marrow [7]. Unfortunately, there was minimal improvement in platelet count after four weeks of romiplostim. 

Our medical team considered the potential risks and benefits of administering epsilon aminocaproic acid (EACA), given the persistent bleeding (epistasis, melena, bleeding from burr hole incision site). Multiple reports in the literature contradict this approach to DIC since blockade of the fibrinolytic system may increase the risk of thrombotic complications [8]. Based on the patient's hyperfibrinolytic state, antifibrinolytic therapy was reasonably effective. Five grams of EACA was administered over 60 min, preventing massive hemorrhage.

## Conclusions

This report highlights the interdisciplinary collaborations between obstetrics, hematology, oncology, neurosurgery, and critical care medicine in managing the multiple complications in DIC. Despite medical care, mortality in patients remains high. New strategies tailored to the underlying pathologic mechanisms are needed, primarily when presenting with infrequent overlapping triggers, such as HELLP syndrome and cancer.
